# Establishment and validation of the autophagy-related ceRNA network in irreversible pulpitis

**DOI:** 10.1186/s12864-023-09363-9

**Published:** 2023-05-19

**Authors:** Ye Wang, Qiuyan Xie, Hongwen Yu, Bangyi Zhou, Xiaolan Guo, Buling Wu, Jiao Hu

**Affiliations:** 1 Shenzhen Stomatology Hospital (Pingshan) of Southern Medical University, Shenzhen, 518118 China; 2 Department of Stomatology, Nanfang Hospital, Southern Medical University, Guangzhou, 510515 China; 3 Changsha Stomatological Hospital, Changsha, 410000 China

**Keywords:** Irreversible pulpitis, Autophagy, ceRNA, lncRNA, Bioinformatics

## Abstract

**Background:**

The molecular mechanisms underlying the onset and progression of irreversible pulpitis have been studied for decades. Many studies have indicated a potential correlation between autophagy and this disease. Against the background of ﻿the competing endogenous RNA (ceRNA) theory, protein-coding RNA functions are linked with long ﻿noncoding RNAs (lncRNAs) and microRNAs (miRNAs). This mechanism has been widely studied in various fields but has rarely been reported in the context of irreversible pulpitis. The hub genes selected under this theory may represent the key to the interaction between autophagy and irreversible pulpitis.

**Results:**

Filtering and differential expression analyses of the GSE92681 dataset, which contains data from 7 inflamed and 5 healthy pulp tissue samples, were conducted. The results were intersected with autophagy-related genes (ARGs), and 36 differentially expressed ARGs (DE-ARGs) were identified. Functional enrichment analysis and construction of the protein‒protein interaction (PPI) network of DE-ARGs were performed. Coexpression analysis was conducted between differentially expressed lncRNAs (DElncRNAs) and DE-ARGs, and 151 downregulated and 59 upregulated autophagy-related DElncRNAs (AR-DElncRNAs) were identified. StarBase and multiMiR were then used to predict related microRNAs of AR-DElncRNAs and DE-ARGs, respectively. We established ceRNA networks including 9 hub lncRNAs (HCP5 and AC112496.1 **↑**; FENDRR, AC099850.1, ZSWIM8-AS1, DLX6-AS1, LAMTOR5-AS1, TMEM161B-AS1 and AC145207.5 **↓**), which were validated by a qRT‒PCR ﻿analysis of pulp tissue from patients with irreversible pulpitis.

**Conclusion:**

We constructed two networks consisting of 9 hub lncRNAs based on the comprehensive identification of autophagy-related ceRNAs. This study may provide novel insights into the interactive relationship between autophagy and irreversible pulpitis and identifies several lncRNAs that may serve as potential biological markers.

## Background

Irreversible pulpitis defined by the American Association of Endodontists (AAE) protocol is characterized by acute pain, increased risks of systematic diseases, and an intensifying economic burden [1, 2]. Pulpotomy and root canal therapy serve as the traditional treatments for irreversible pulpitis, and some dentists prescribe antibiotics to relieve pain [3, 4]. Microbiological and histological research has proven that microbial invasion, inflammation, and microabscesses are observed in all teeth with irreversible pulpitis [5]. Thus, the molecular mechanisms underlying these bioprocesses are worthy of further investigation. To date, several studies have revealed the interactions between irreversible pulpitis and various inflammatory factors, such as the Toll-like receptors interleukin-8 (IL-8) and interleukin-6 (IL-6) [6]. In addition, bioinformatic analyses have shown that various potential mechanisms, including cell adhesion and lymphocyte activation, interact with the disease [7].

Autophagy plays a key role in the maintenance of cellular homeostasis by contributing to cellular metabolism, innate immunity, and cell survival [8, 9]. Numerous lines of evidence have shown a significant relationship between autophagy and inflammation in infections, cancer, metabolic disorders, and liver diseases [10, 11]. Interestingly, several previous studies have suggested that autophagy is related to pulpitis. Yin et al. proved that JMJD3 participates in the onset and progression of pulpitis by reducing the di- and tri-methylation of H3K27 and thus regulates key autophagy genes [12]. Since most researchers have indicated that autophagy is closely connected with oral diseases such as periodontitis and pulpitis, the crosstalk between autophagy and irreversible pulpitis requires comprehensive investigation.

Shifting from tissue studies to the gene level, the mechanism underlying the crosstalk between autophagy and irreversible pulpitis remains unclear. In recent years, lncRNA, a type of noncoding RNA (ncRNA) with a length of more than 200 bp, has received increasing attention due to its roles in numerous biological processes [13]. Considering the noncoding nature of lncRNAs, scientists have focused on their role in epigenetic regulation, particularly on the competing endogenous RNA (ceRNA) theory. This theory links protein-coding RNA functions with lncRNA functions and reveals how lncRNAs influence the progression of a series of diseases [14]. Studies have proven that lncRNAs participate in cancer, autoimmune deficiency, and pulmonary disease [15–17]. Limited studies have investigated ceRNAs in pulpitis. Lei et al. constructed a ceRNA network for the selection of potential marker genes for the disease [18]. Xi et al. identified several lncRNAs with the aim of revealing the relationship between inflammation and pulpitis [19]. However, to our knowledge, the interaction between autophagy and irreversible pulpitis against the background of the ceRNA theory remains unknown.

In this study, we aimed to elucidate the interaction between autophagy and irreversible pulpitis based on the ceRNA theory associated with lncRNAs. By considering differentially expressed genes (DEGs) and selecting those intersecting with autophagy markers, ceRNA networks were constructed based on differentially expressed lncRNAs (DElncRNAs) and selected differentially expressed autophagy-related genes (DE-ARGs). In addition, we identified several hub lncRNAs in the ceRNA networks as potential biological markers of irreversible pulpitis.

## Results

### Data filtering

**Fig. 1:**
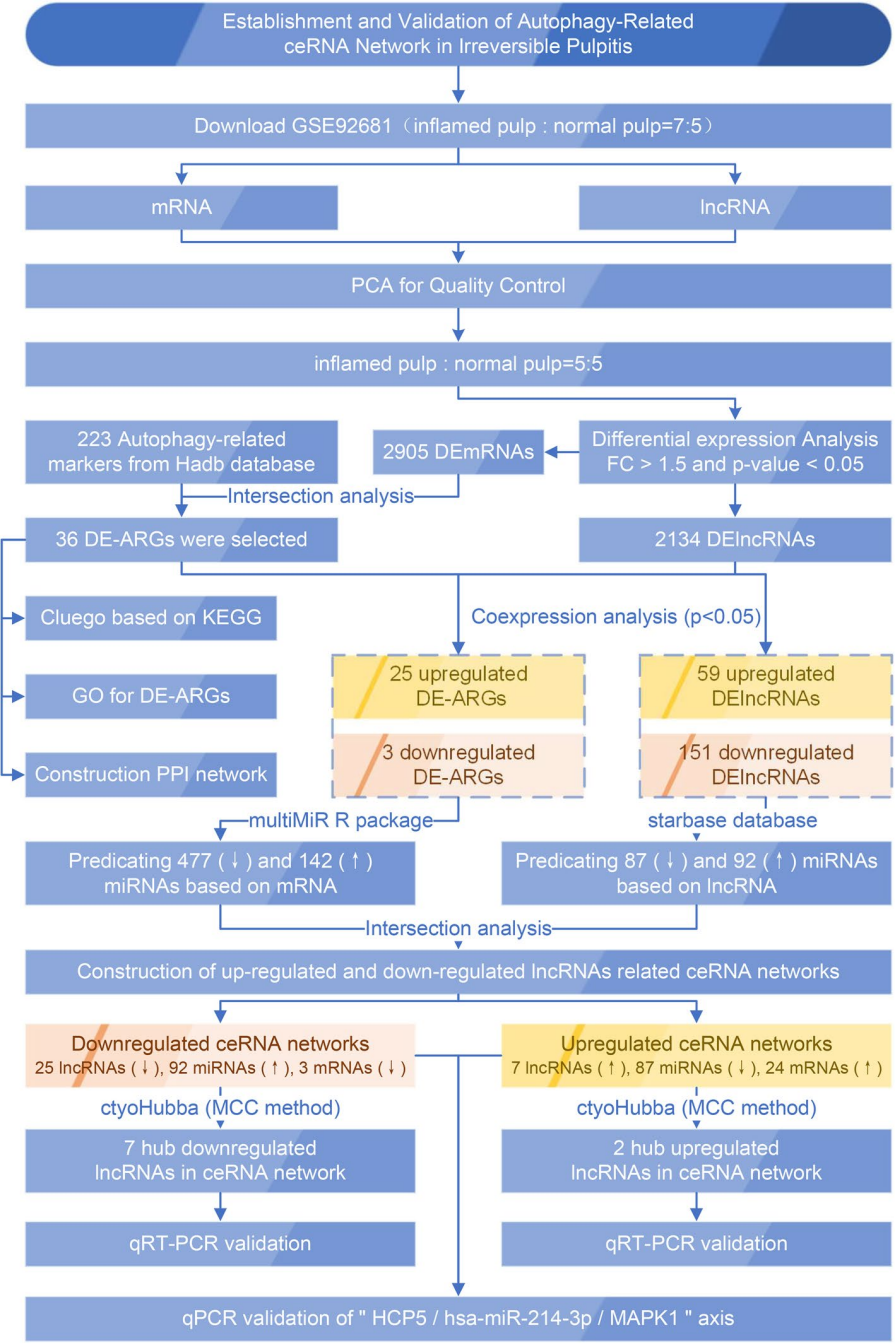
Workflow of the study

**Fig. 2:**
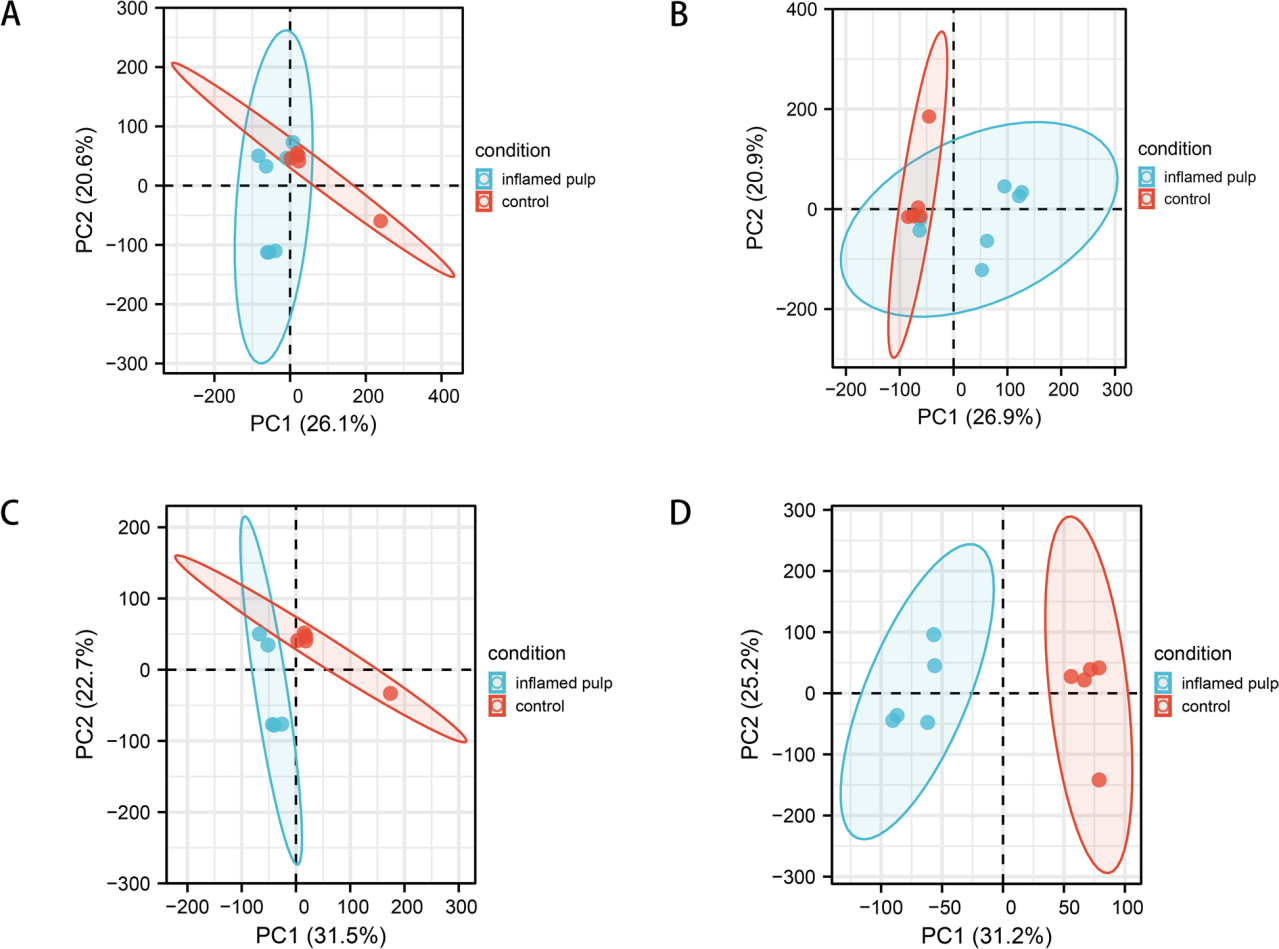
Quality control. **A** Principal component analysis (PCA) of lncRNAs. **B** PCA of mRNAs. **C** PCA of lncRNAs after quality control. **D** PCA of mRNAs after quality control

Figure 1 summarizes the workflow of the current investigation. Principal component analysis (PCA) indicated that GSM2434473 and GSM2434475 most likely originated from pulpitis tissues that were incorrectly included in the control group. Figure 2A and B shows the clustergrams of lncRNAs and mRNAs that have not been validated. Figure 2C and D show the results after removing nonnormal samples (GSM2434473 and GSM2434475). After validation, the dispersion between the control and pulpitis groups was markedly improved in 5 control (GSM2434481, GSM2434482, GSM2434483, GSM2434484, and GSM2434480) and 5 pulpitis samples (GSM2434474, GSM2434476, GSM2434477, GSM2434478 and GSM2434479). Thus, the above 10 samples were selected for further analysis.

### Identification of DE-ARGs

**Fig. 3:**
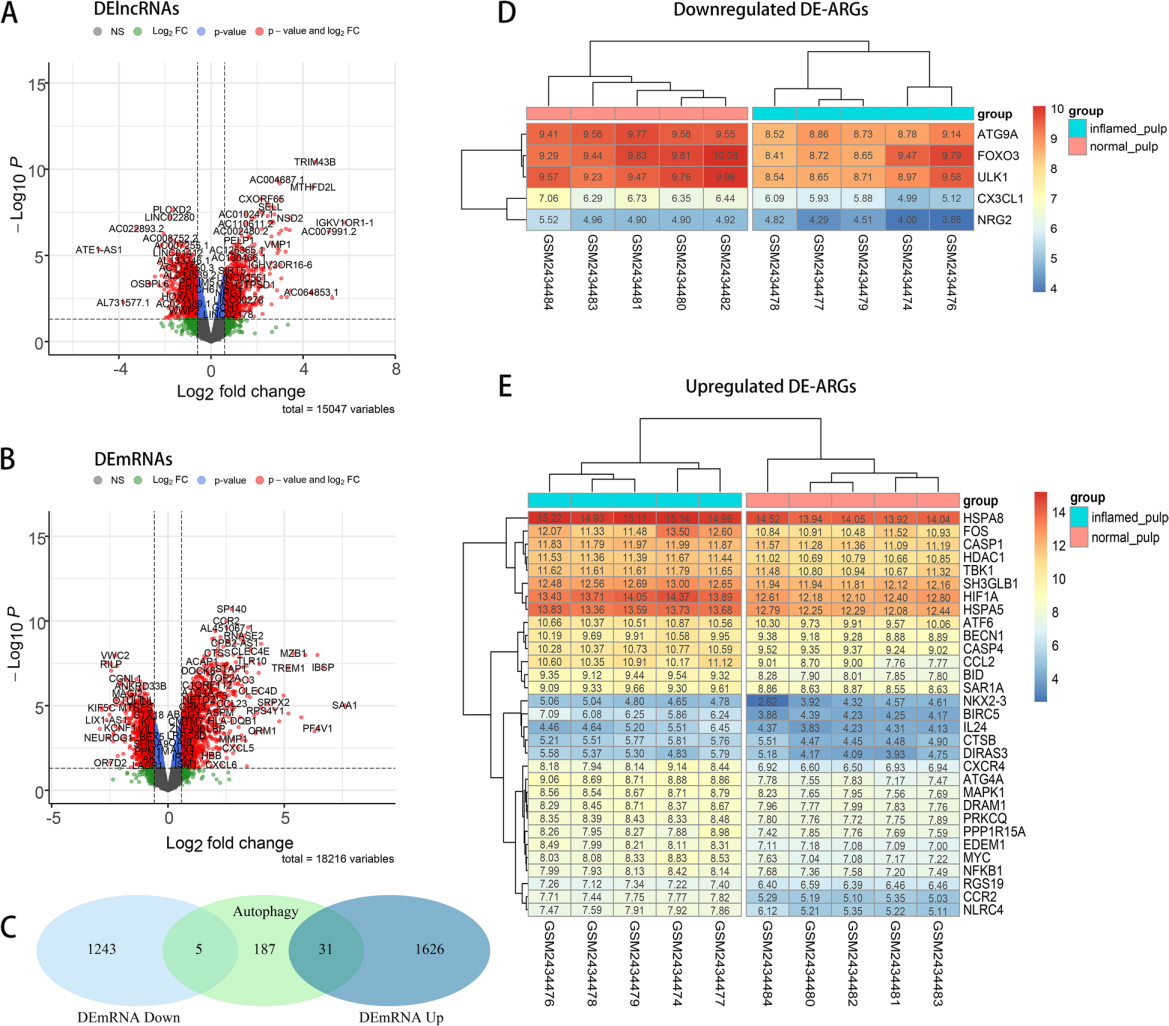
Identification of DE-ARGs and DE-lncRNAs. **A** Volcano plot of the results from the analysis of DElncRNAs. **B** Volcano plot of the results from the analysis of the DEmRNAs. **C** Venn diagram of DEGs and ARGs. **D** Heatmap of downregulated DE-ARGs in the samples. **E** Heatmap of upregulated DE-ARGs in the samples

The volcano plots shown in Fig. 3A and B revealed the differential expression of lncRNAs and mRNAs between the two groups. Based on the cut-offs of a *p* value < 0.05 and an FC > 1.5, DEGs were identified, and 893 upregulated and 1241 downregulated lncRNAs and 1657 upregulated and 1248 downregulated mRNAs were recorded. A total of 223 marker genes for autophagy were obtained from the HAdb database. Figure 3C shows the genes presenting correlations between the identified autophagy markers and DEGs in a Venn diagram. Accordingly, 36 DE-ARGs, including 5 upregulated and 31 downregulated ARGs, were screened out. As shown in Fig. 3D and E, the expression levels of the 36 DE-ARGs in 10 human pulp tissue samples were visualized in hot plots.

### Functional enrichment and protein‒protein interaction (PPI) network analyses

**Fig. 4:**
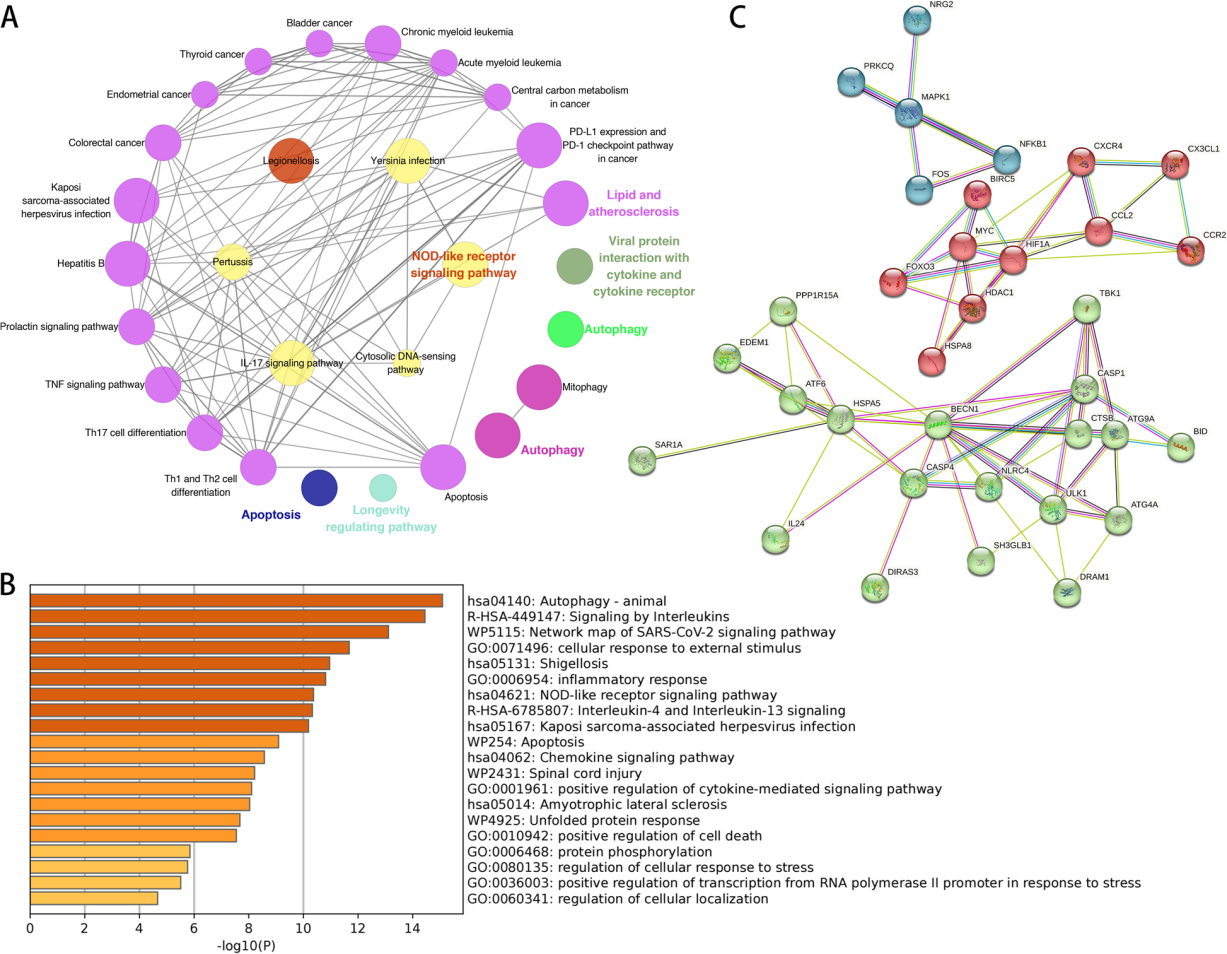
Functional enrichment analysis of DE-ARGs. **A** ClueGO analysis of DE-ARGs. **B** Enrichment analysis of DE-ARGs based on the Metascape database. **C** 3 PPI subnetworks of DE-ARGs divided by k means clustering

The functional network based on all the DE-ARGs against the background of the Kyoto Encyclopedia of Genes and Genomes (KEGG) database [20, 21] and the estimated criterion of *p* value < 0.01 is shown in Fig. 4A. Numerous biochemical processes, including autophagy, apoptosis, the NOD-like receptor signalling pathway, and the longevity regulating pathway, were enriched. Figure 4B depicts bar charts of the enrichment analysis results based on the Metascape database. In this list, most of the genes were found to be involved in the response to stimuli among the parental categories but were related to subcategories of autophagy, signalling by interleukins, the SARS-CoV-2 signalling pathway, and the cellular response to external stimuli. To explore the mechanisms underlying the roles of DE-ARGs in the enriched pathways, we next focused on the interactions between proteins. The PPI network was built based on the STRING database, and DE-ARGs with no interactions were removed, resulting in a total of 36 nodes and 111 edges with an average node degree of 6.17. As shown in Fig. 4C, we divided the PPI network of DE-ARGs into 3 subnetworks by k-means clustering. Within the subnetworks, BECN1, HIF1A, HSPA5, MYC, CASP1, HSPA8, FOS, FOXO3 and NFKB1 may play an essential role in the whole landscape with the greatest number of connections (node degree ≥ 10). Proteins involved in the molecular mechanism were identified for further discussion.

### Coexpression analysis

**Fig. 5:**
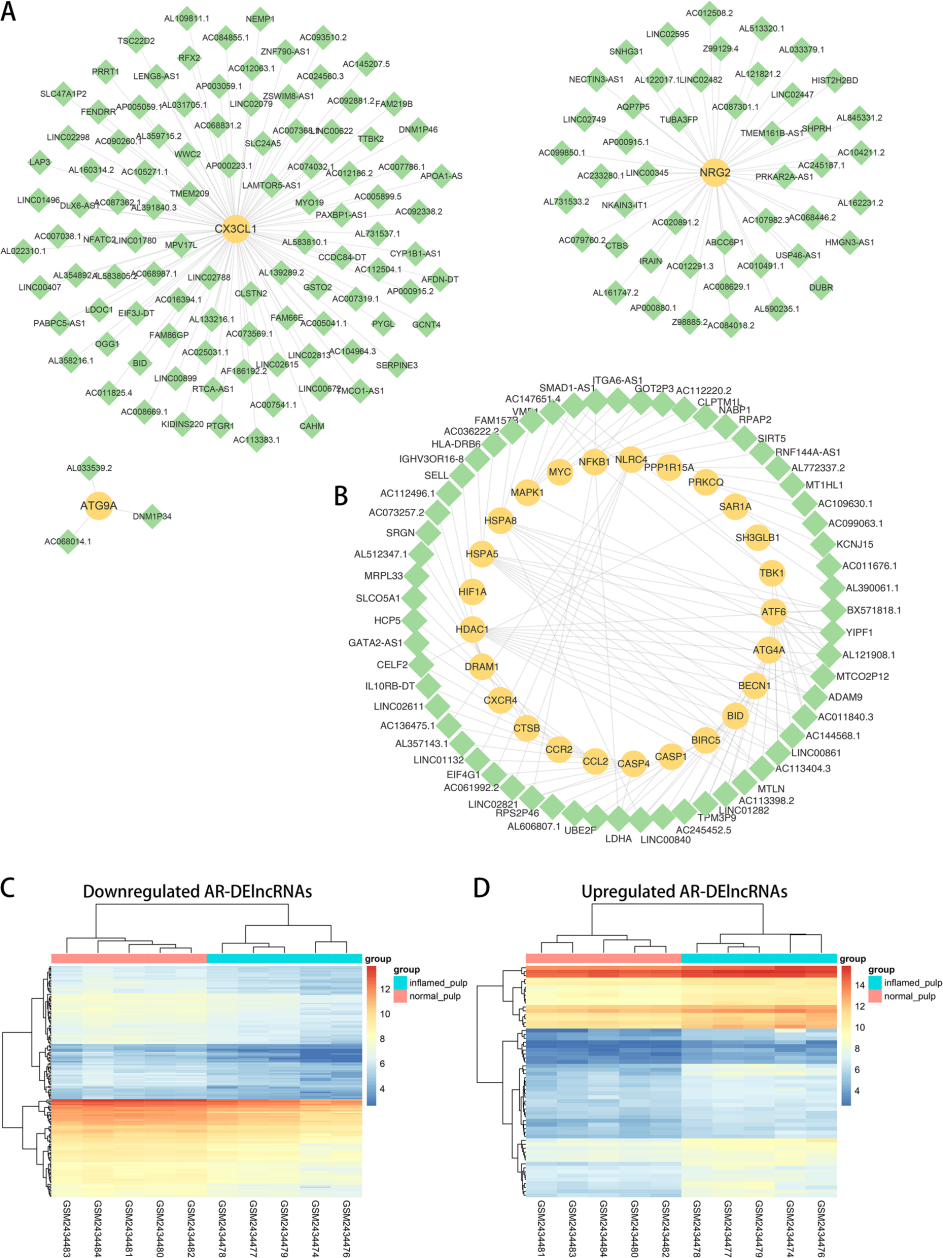
Coexpression analysis of DE-ARGs and DElncRNAs. **A** Coexpression network of downregulated DElncRNAs/DE-ARGs (lncRNA: green; mRNA: yellow). **B** Coexpression network of upregulated DElncRNAs\DE-ARGs (lncRNA: green; mRNA: yellow). **C** Heatmap of the expression of 151 downregulated AR-DElncRNAs in the downregulated coexpression network. **D** Heatmap of the expression of 59 upregulated AR-DElncRNAs in the upregulated coexpression network

Figure 5A shows the coexpression network of the downregulated group (downregulated DElncRNAs and downregulated DE-ARGs), involving 151 DElncRNAs and 3 DE-ARGs. Additionally, the network of upregulated DElncRNAs and upregulated DE-ARGs which included 59 DElncRNAs and 25 DE-ARGs is shown in Fig. 5B. AR-DElncRNAs were selected from the two coexpression networks, and their expression levels among the 10 human pulp tissue samples are shown in Fig. 5C and D.

### Construction of lncRNA-related ceRNA networks

**Fig. 6:**
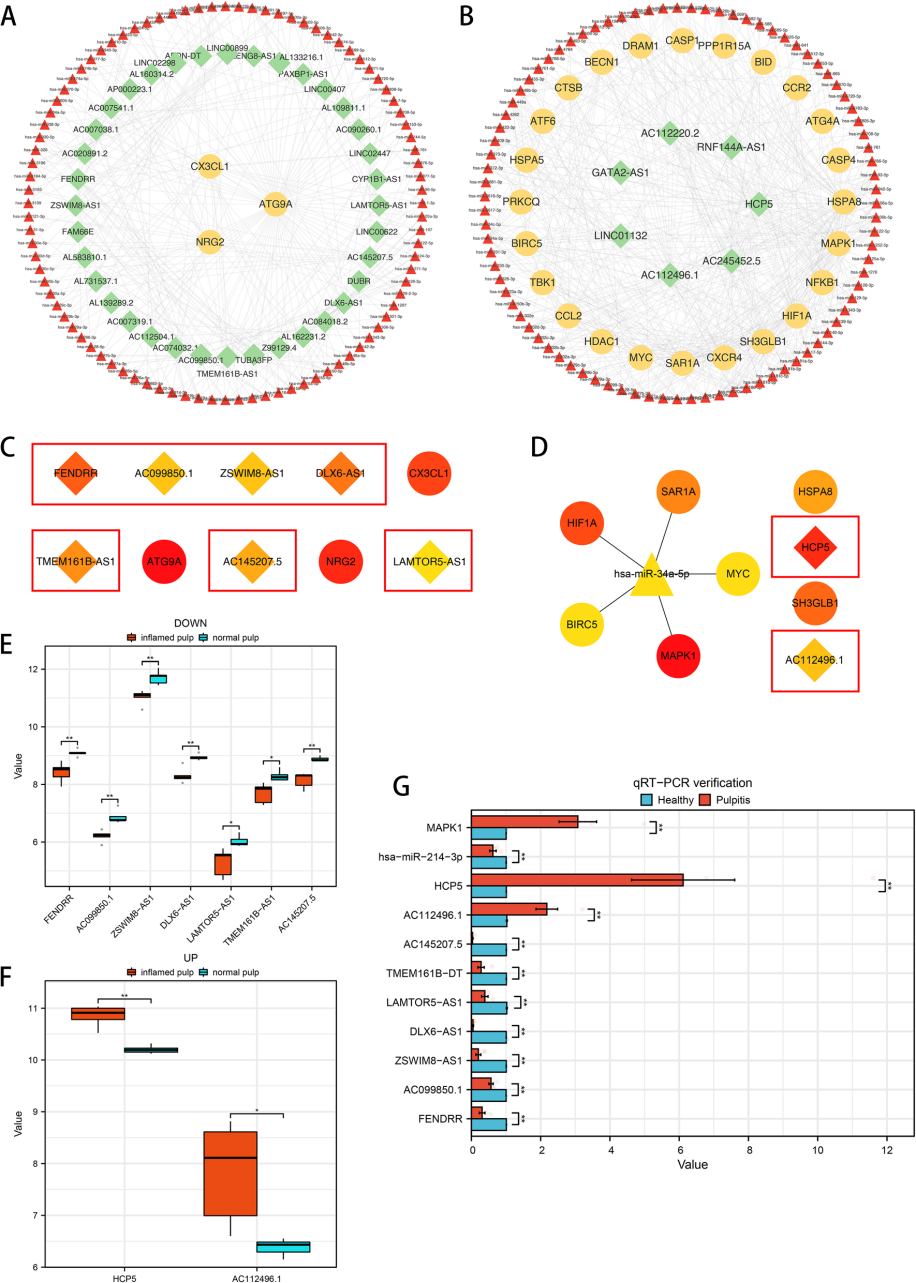
Construction of ceRNA networks. **A** Downregulated lncRNA-related ceRNA networks. **B** Upregulated lncRNA-related ceRNA networks. **C** Hub nodes of ceRNAs in downregulated group by MCC method (lncRNA: rhombus, mRNA: circle, miRNA: triangle). **D** Hub nodes of ceRNAs in upregulated group by MCC method (lncRNA: rhombus, mRNA: circle, miRNA: triangle). **E** Boxplot of the expression levels of 7 hub downregulated lncRNAs selected from the microarray dataset. **F** Boxplot of the expression levels of 2 hub upregulated lncRNAs selected from the microarray dataset. **G** Gene expression levels in ﻿human pulp tissues evaluated ﻿by qRT‒PCR (*n* = 5), ﻿* *P* < 0.05

Taking the upregulated group as an example, 87 microRNAs (miRNAs) related to AR-DElncRNAs and 477 miRNAs associated with DE-ARGs were identified. The intersection between the two clusters of miRNAs, including 87 miRNAs, was chosen for the following analysis. Simultaneously, 92 intersecting miRNAs from the downregulated group were found among 92 AR-DElncRNA-related miRNAs and 142 DE-ARG-related miRNAs. Ultimately, based on the intersecting miRNAs, associated AR-DElncRNAs and DE-ARGs, ceRNA networks of the downregulated lncRNAs and upregulated lncRNAs were constructed, as shown in Fig. 6A and B, respectively. CytoHubba analysis with the maximal clique centrality (MCC) method was used to identify hub lncRNAs in the two networks, which included FENDRP, AC099850.1, ZSWIM8-AS1, DLX6-AS1, TMEM161B-AS1, AC145207.5 and LAMTOR5-AS1 in the downregulated network and HCP5, AC112496.1 in the upregulated network, as shown in Fig. 6C and D. In addition, the expression levels of the nine hub lncRNAs in the dataset are shown in box plots in Fig. 6E and F.

### Quantitative real-time polymerase chain reaction (qRT-PCR) validation

﻿To further confirm the expression levels of the abovementioned nine hub lncRNAs selected from the ceRNA networks, their expression levels in five ﻿human ﻿inflamed pulp tissues and five normal pulp tissue samples were evaluated by ﻿qRT‒PCR (Fig. 6G), and ﻿the validation results were consistent with the data from the microarray analysis. Additionally, the expression levels of hsa-miR-214-3p and MAPK1 which were considered to have the highest correlation with HCP5 within the ceRNA networks in five human inflamed pulp tissues and five normal pulp tissue samples were showed in Fig. 6G. The expression level results of HCP5, hsa-miR-214-3p and MAPK1 matched the corresponding changes of ceRNA network.

## Discussion

The microbial-induced destruction of immune homeostasis is involved in the onset and progression of pulpitis [22]. Clinically, this condition develops to an irreversible state through gradual demineralization and damage to enamel and dentine caused by caries [23]. Accumulating studies have proven the contribution of the inflammatory response to irreversible pulpitis, including the significant upregulation of cytokines such as IL-8, TNF-α, and RAGE [24]. From another point of view, autophagy is considered a core biochemical mechanism in both physiological equilibrium maintenance and disease occurrence, particularly in inflammatory diseases [25]. Qi et al. showed that the expression of ARGs is markedly increased in rat disease models [26], suggesting the influence of autophagy on irreversible pulpitis. Moreover, ATG9A and ATG4A, which were found to be upregulated in the abovementioned study, were also identified in our PPI network. Since ARGs have been proven to be biomarkers of autophagy [22], the interaction between autophagy and irreversible pulpitis is worthy of further investigation. Thus, the current research focused on gene expression and searches for specific molecules or pathways to elucidate the potential mechanism of autophagy in irreversible pulpitis.

Ever since the blueprint of the human genome was provided based on sequencing techniques, much attention has been given to protein-coding genes. However, noncoding RNAs, which account for nearly 98% of the total genome, have been shown to carry out biological functions that vary from RNA processing and translation to DNA synthesis or genome rearrangement [27, 28]. Many researchers have systematically shown that lncRNAs are involved in autophagy processes and related disease progression [29, 30]. Additionally, the ceRNA hypothesis suggests that lncRNAs can competitively bind to specific miRNAs and thereby regulate miRNA-related mRNAs and downstream pathways [31]. The network identified in the current study might shed light on the interactions among lncRNAs, mRNAs, and miRNAs to reveal the potential role played by lncRNAs in disease mechanisms. Numerous studies have drawn attention to this modification method. Lei et al. identified the lncRNA PVT1 as a therapeutic target in pulpitis via a ceRNA network, whereas other studies identified XIST, MIR155HG, and LINC00630 as targets for further investigation [18]. Bian et al. discussed the relationship between periodontitis and autophagy based on a ceRNA network [32] and selected hsa-miR-671-5p as the core of the interactive mechanism and a therapeutic marker. Interestingly, the same miRNA was identified in our network, indicating its potential role in pulpitis. According to current investigations, the ceRNA hypothesis for pulpitis is limited, and the crosstalk between autophagy and pulpitis remains unclear. Therefore, our research aimed to identify innovative biomarkers by selecting DEGs and analysed the ceRNA network to reveal the mechanism underlying the interaction between irreversible pulpitis and autophagy.

With the advancement of bioinformation analyses in various fields of research, fire-new insights are provided into genetic information underlying the physical and pathological mechanism. A novel computational method defined as “GCNCRE” is explored to predict potential human lncRNA-miRNA interactions [33]. Compared with our research, this theory is more accurate based on accumulative calculations and more complicated. Meanwhile, the network distance analysis and a logistic matrix factorization with neighbourhood regularized aiming at lncRNA-related miRNA predictions may provide us with more accurate methods for miRNA predictions [34, 35]. Besides, more information will be discovered by a deep learning method for metabolite-disease associations prediction [36]. It may serve as the supplement to gene markers predictions via investigation of specific metabolism mechanism within diseases. After identification of gene markers, the specific molecular fingerprint features can be analysed by a deep learning predictive model named DMFGAM [37]. However, the applications of these bioinformation methods in pulpitis are limited. More investigations are needed for this field to enrich both mechanism methods of the disease and the practicability of these methods.

Nine hub lncRNAs, including HCP and AC112496.1, which were upregulated, and FENDRR, AC099850.1, ZSWIM8-AS1, DLX6-AS1, LAMTOR5-AS, TMEM161B-AS1, and AC145207.5, which were downregulated, were selected from the ceRNA networks. Based on previous investigations, HCP5, FENDRR and DLX6-AS1 were considered to be related to autophagy, but only these three RNAs and four others have been analysed previously. As shown by existing evidence, HCP5 negatively regulates miR-214-5p to influence bioprocesses such as autophagy and apoptosis in pancreatic cancer via the miR-214-5p/HDGF axis [38]. Other studies identified HCP5 as the core regulator of autophagy through lncRNA‒mRNA coexpression analysis and revealed that HCP5 interacts with the miR-93-5p/HMGA2 axis and modifies the activity of the AKT/mTOR signalling pathway. Another hub lncRNA, FENDRR, may inhibit autophagy by binding PRC2 and downregulating ATG7 [39]. As revealed by clinical research, the FENDRR levels are significantly decreased in peripheral blood derived from patients with inflammatory periodontal tissue compared with that from healthy individuals [40]. Considering the interrelation of periodontal and pulp tissue, FENDRR may be involved in the lesions of both tissues. As a final example, the upregulation of miR-193b-3p or the downregulation of HOXA1 decrease the DLX6-AS1 levels, which hinders cell proliferation and promotes autophagy [41]. Accordingly, the hub lncRNAs selected in the current study have been proven to be related to cell autophagy through various pathways. Moreover, this study provides the first identification of these lncRNAs as core components of the interaction between autophagy and pulpitis, and this finding is worthy of further in vivo or in vitro studies.miRNAs are involved in epigenetic mechanisms and serve as the bridge for lncRNAs to regulate mRNAs according to the ceRNA hypothesis. For example, hsa-miR-214-3p participates in modulating cell autophagy through the HCP-mediated miR-214-3p/HDGF axis, as previously discussed [38]. As shown by our ceRNA network, HCP5 is closely related to several mRNAs, such as CCL2 and MAPK1. MAPK1 has been extensively studied and proven to be involved in inflammation processes and to simultaneously participate in ATG7-induced cell autophagy [42]. Surprisingly, we discovered that CCL2 inhibits cell autophagy and serves as a potential biomarker for pulpitis [43, 44]. Similar to the PPI network of Chen et al., we also screened CCL2 as the core, thereby indicating that CCL2 is the focus of the interactive mechanism of autophagy and irreversible pulpitis. Accordingly, HCP5 may be associated with autophagy and irreversible pulpitis. Interestingly, the ceRNA networks in our study show that HCP5 regulates MAPK1 through hsa-miR-214-3p. The experiment conducted by Lee et al. proved that MAPK1 might be associated with antioxidant activity in LPS-induced dental pulp tissue [45]. To summarize the previous discussion, more attention should be given to the crosstalk among HCP5, hsa-miR-214-3p, and MAPK1 since they have been indicated to function in autophagy or pulpitis. Our results of qRT-PCR also showed that the expression level of HCP5, hsa-miR-214-3p and MAPK1 matched the corresponding changes based on the ceRNA theroy. HCP5 may target hsa-miR-214-3p and compete with MAPK1, thus playing a role in cell autophagy and the modulation of irreversible pulpitis.

Several limitations of our study need to be mentioned. Initially, a differential expression analysis of miRNA was not included in the study due to the lack of irreversible pulpitis-related miRNA maps. In further investigations, we will focus on the specific mechanisms of selected molecules in irreversible pulpitis with the aim of providing potential therapeutic targets to the research field.

## Conclusions

﻿Overall, we screened nine hub lncRNAs based on ceRNA networks as candidate regulators and thus provide a novel reference for the subsequent exploration of the link between autophagy and irreversible pulpitis.

## Materials and methods

### Data source and processing

Microarray data from GSE92681, which includes data from seven human pulpitis samples and five control samples of normal pulp tissues, were obtained from the National Center for Biotechnology Information (NCBI) website and its Gene Expression Omnibus (GEO) database (https://www.ncbi.nlm.nih.gov/geo/). All of the samples were analysed using the GPL16956 platform (Agilent-045997 Arraystar human lncRNA microarray V3) [40]. Furthermore, factoextra in R was used for cluster analysis of all the samples, and high-quality samples (5 pulpitis and 5 healthy samples) were screened for PCA with the factoextra and FactoMineR R packages. Boxplots and clustergrams were created for visualization.

### DEG and lncRNA analysis

The total datasets from 10 screened samples were sorted into mRNA and lncRNA subsets. The Limma R package was then applied separately to each subset for the analysis of DEGs and DElncRNAs between pulpitis pulp samples and healthy pulp samples using the cut-off criteria of a *p* value < 0.05 and a |log2-fold change (FC)|> 1.5. The R package EnhancedVolcano was used to visualize the abovementioned upregulated and downregulated DEGs.

### Identification of DE-ARGs

A total of 223 ARGs were acquired from the HAdb database (http://www.autophagy.lu/index.html). Thereafter, the DEGs were separated into upregulated and downregulated groups, and DE-ARGs were defined based on intersection analysis between ARGs and upregulated or downregulated DEGs. Venn diagrams were drawn to reveal the overlap between the DEGs and ARGs. Moreover, Pheatmap in the R package was applied to generate heatmap plots, which reveal the expression levels of DE-ARGs in the 10 pulp samples from GSE92681.

### Functional enrichment analyses of DE-ARGs

To investigate the biological functions and pathways of DE-ARGs, the ClueGO function in Cytoscape 3.9.1 was used with the evaluation criteria of an adjusted *p* value < 0.01, and the KEGG database was employed for pathway enrichment analysis. Additionally, the Metascape database was chosen for enrichment analysis, and the results were visualized in enriched bar graphs. The PPI network was constructed based on the String database (https://cn.string-db.org/), in which the protein‒protein interactions of DE-ARGs were summarized as the background; a confidence value > 0.4 was used as the cut-off value; and nodes that interacted with no proteins were removed. Subsequently, we divided the PPI network of DE-ARGs into subnetworks with a k-means clustering algorithm.

### Coexpression analysis of DE-ARGs and DElncRNAs

To reveal the correlation between DE-ARGs and DElncRNAs, a coexpression analysis was performed with the Psych R package based on the identified upregulated or downregulated genes. A relevance value > 0.97 and *p* value < 0.05 were set as the thresholds, and the Ggcorrplot R package was used for visualization. After removing DElncRNAs with no interacting DE-ARGs, Cytoscape 3.9.1 was used to visualize the coexpression network. The expression levels of extracted autophagy-related DElncRNAs (AR-DElncRNAs) in 10 pulp samples from GSE92681 were then revealed using the Pheatmap R package.

### Construction of the ceRNA network

The starBase and multiMiR R packages, which are tools for predicting miRNA binding sites, were used to estimate the miRNAs with which AR-DElncRNAs and DE-ARGs may interact. An intersection analysis combining miRNA/AR-DElncRNAs and miRNA/DE-AGRs was then conducted to build ceRNA networks of upregulated and downregulated lncRNAs, respectively. Visualization of the results was performed with Cytoscape 3.9.1. To further elucidate the relevant molecular mechanisms, the cytoHubba R package was used with the MCC method to screen out the top nine hub lncRNAs among the two ceRNA networks. Thereafter, the expression levels of hub lncRNAs were shown in box plots using ggplot2 in the R package.

### qRT‒PCR validation of hub lncRNAs

﻿qRT‒PCR was performed ﻿to further verify the levels of the nine hub lncRNAs of the ceRNA networks in five ﻿﻿inflamed human pulp tissues and five normal pulp tissue samples. In addition, to further verify the interactions within the ceRNA network, “HCP5/ hsa-miR-214-3p / MAPK1” axis was chosen for qPCR validation. Specimen collection and research were carried out in accordance with relevant guidelines and regulations and approved by the Ethics Committee of Southern Medical University (NFEC-2023–079). Inflamed pulp tissues were extracted from teeth diagnosed with irreversible pulpitis in accordance with the endodontics diagnosis system of the AAE. Normal pulp samples were collected from healthy third molars or teeth extracted for orthodontic purposes. Patients who had a compromised immune system or were taking medications known to influence the immune response were excluded. Any teeth that suffered from periodontitis were excluded from the study. Informed consent was obtained from all the patients.

Total tissue RNA was extracted with a Quick-RNA Miniprep Kit (ZYMO Research, Irvine, CA, USA) after pretreatment with TRIzol (Life Technologies) according to the manufacturer’s instructions, as previously reported [41]. One microgram of total RNA was employed for reverse transcription using M-MLV Reverse Transcriptase (Thermo Scientific, Waltham, MA, USA) and was detected using PowerUp SYBR Green Master Mix (Thermo Scientific) and a Bio-Rad iQ5 thermal cycler (Bio-Rad Laboratories, Hercules, CA, USA). Differences of LncRNA and mRNA in expression were evaluated by the comparative cycle threshold method using GAPDH as a control. And U6 was used as the internal reference gene for miRNA. The primer sequences are listed in Table 1.

**Table 1:** Sequences of the primers used in the qRT‒PCR experiments

Primer name	Sequence 5’-3’
GAPDH Forward	GGAGCGAGATCCCTCCAAAAT
GAPDH Reverse	GGCTGTTGTCATACTTCTCATGG
AC112496.1 Forward	CAGCCACTCATCCCTAATGTCT
AC112496.1 Reverse	TTGTTGCTGCCTTCATCAGGT
HCP5 Forward	ATCCAGACCTGGGCAGATTAC
HCP5 Reverse	TTCTCTCCTTCTGCCCATCAC
AC099850.1 Forward	GTCAATATAAAGGGGACTCCTCCA
AC099850.1 Reverse	AAAGTTTTGGCGTGGGTTGG
AC145207.5 Forward	TGACTGGCCAAGCATTTGGT
AC145207.5 Reverse	GCTACATGATCACAGACAAGCTG
DLX6-AS1 Forward	TGTTTTTGGCCATTGCGGAG
DLX6-AS1 Reverse	AGCTCCCCTTTCCTATGCTCT
FENDRR Forward	GCGCACAGACCCAGGATTT
FENDRR Reverse	GGGCAGAGCTGGTTTTGACA
LAMTOR5-AS1 Forward	CCCTAGTGCAACAGAGCATGA
LAMTOR5-AS1 Reverse	CCAAGGGGAATGTGGGAGTC
TMEM161B- AS1 Forward	GCCACGCAGAGGTGAAGATA
TMEM161B- AS1 Reverse	TTTAAACTGTGCTGCGCCTG
ZSWIM8-AS1 Forward	CACAACTGCAGTCAGGTCAAAT
ZSWIM8-AS1 Reverse	GGCCTGCACAACTTTGTTTCT
MAPK1 Forward	TACACCAACCTCTCGTACATCG
MAPK1 Reverse	CATGTCTGAAGCGCAGTAAGATT
hsa-miR-214-3p Forward	GCACAGCAGGCACAGACA
hsa-miR-214-3p Reverse	GAGCAGGGTCCGAGGT
U6 Forward	CTCGCTTCGGCAGCAC
U6 Reverse	AACGCTTCACGAATTTGCG
hsa-miR-214-3p for reverse transcription	GTCGTATCCAGTGCAGGGTCCGAGGTAT
	TCGCACTGGATACGACACTGCC
U6 for reverse transcription	AAAATATGG

### Statistical analysis

qRT‒PCR data were processed with GraphPad Prism software version 8.0.1 (San Diego, CA, USA), and the means ± standard deviations (means ± SDs) are presented for the quantitative data. Data from two groups were assessed by t tests. Data involving more than two groups were assessed by one-way analysis of variance (ANOVA), and the level of significance was set to a *P* value < 0.05. The statistical analyses of the microarray data were performed using R software (Version 4.1.2, https://www.r-project.org/).

## Data Availability

A publicly available dataset (GSE92681) was reanalysed in this study. All raw data generated during the re-analysis and by qRT‒PCR experiments are available in the Jianguoyun Repository (https://www.jianguoyun.com/p/DWhg7yYQt-T1ChjWuPMEIAA).

## Abbreviations

ceRNA: Competing endogenous RNA
lncRNA: Long noncoding RNA
miRNA: MicroRNA
ARG: Autophagy-related gene
DE-ARG: Differentially expressed autophagy-related gene
PPI: Protein‒protein interaction
DElncRNA: Differentially expressed lncRNA
AR-DElncRNA: Autophagy-related differentially expressed lncRNA
AAE: American association of endodontists
IL-8: Interleukin-8
IL-6: Interleukin-6
ncRNA: Noncoding RNA
DEG: Differentially expressed gene
PCA: Principal component analysis
KEGG: Kyoto encyclopedia of genes and genomes
qRT‒PCR: Quantitative real-time polymerase chain reaction
NCBI: National center for biotechnology information
GEO: Gene expression omnibus
ANOVA: Analysis of variance
